# Case Report: Successful treatment of generalized pustular psoriasis with concomitant renal insufficiency using spesolimab: a report of two cases

**DOI:** 10.3389/fimmu.2026.1880139

**Published:** 2026-10-05

**Authors:** You Xie, Feng Cheng, Zilu Qu, Lei Hu, Yun Xia, Liuqing Chen, Jinbo Chen

**Affiliations:** 1 Department of Dermatology, Traditional Chinese and Western Medicine Hospital of Wuhan, Tongji Medical College, Huazhong University of Science and Technology, Wuhan, Hubei, China; 2 Hubei Province and Key Laboratory of Skin Infection and Immunity, Wuhan, Hubei, China; 3 Wuhan No 1 Hospital, Wuhan, Hubei, China

**Keywords:** biologics, generalized pustular psoriasis, renal insufficiency, spesolimab, targeted therapy

## Abstract

Generalized pustular psoriasis (GPP) is a rare, severe, and potentially life-threatening neutrophilic dermatosis. Management focuses on rapid control of acute flares and prevention of recurrence. Advances in understanding the central role of interleukin-36 (IL-36) signaling in GPP pathogenesis have shifted the treatment paradigm from traditional systemic immunosuppressants toward targeted biologic therapies. This case report details two patients with GPP and comorbid renal insufficiency who achieved rapid and significant clinical improvement with spesolimab, an IL-36 receptor antagonist. Both patients presented with acute GPP flares and moderate renal impairment. Following a single 900 mg intravenous infusion of spesolimab, skin lesions and systemic symptoms resolved promptly, with disease stability maintained during follow-up. This report provides preliminary evidence supporting the efficacy and safety of spesolimab in this special patient population.

## 1 Introduction

Generalized pustular psoriasis (GPP) is a rare, severe autoimmune dermatosis characterized by widespread sterile pustules on erythematous skin (1). It is often accompanied by systemic manifestations such as high fever, electrolyte disturbances, and hypoalbuminemia (2). The disease has an acute onset and tends to follow a severe course. While its pathogenesis is not yet fully understood, the IL-36-mediated cytokine axis plays a dominant role in its development (3). Current research indicates that GPP was closely associated with multiple susceptibility genes including *IL36RN* (4), *CARD14* (5), *AP1S3* (6), *MPO* (7), *SERPINA3* (8) and *MEFV* (9), among which IL36RN serves as the core pathogenic gene dominating the IL-36 inflammatory pathway. Spesolimab, a humanized monoclonal antibody targeting the IL-36 receptor, has demonstrated remarkable efficacy in treating GPP flares (10). Although emerging case reports, such as those describing the use of spesolimab in patients with HIV, have begun to provide preliminary insights (11), clinical data regarding its use in patients with significant comorbidities, particularly renal insufficiency, remain limited. We present two cases of GPP with moderate renal insufficiency successfully managed with spesolimab.

## 2 Case presentation

### 2.1 Case 1

A 61-year-old male presented with a 6-day history of exacerbating generalized erythema and pustules following oral antibiotic therapy for respiratory symptoms. He had a 30-year history of biopsy-confirmed GPP (**Figures 1A, B**). The patient reported the initial onset of generalized erythema 30 years ago, diagnosed as plaque psoriasis at an outside hospital. Treatment with subcutaneous corticosteroid injections (specific timing and dosage unknown) resulted in temporary control with subsequent recurrence. One year later, the rash worsened with pustule formation, leading to evaluation at our hospital. Histopathological examination confirmed pustular psoriasis. The patient received subcutaneous and oral corticosteroid therapy for 2 years (details unspecified), discontinued after rash resolution. During subsequent annual flare-ups, the patient was managed at outside hospitals with oral traditional Chinese medicine and topical antifungal ointment (details unspecified), achieving only transient control. Approximately one year ago, the patient initiated ustekinumab therapy at our outpatient clinic, administered every three months, with partial disease control until the current exacerbation.

**Figure 1:**
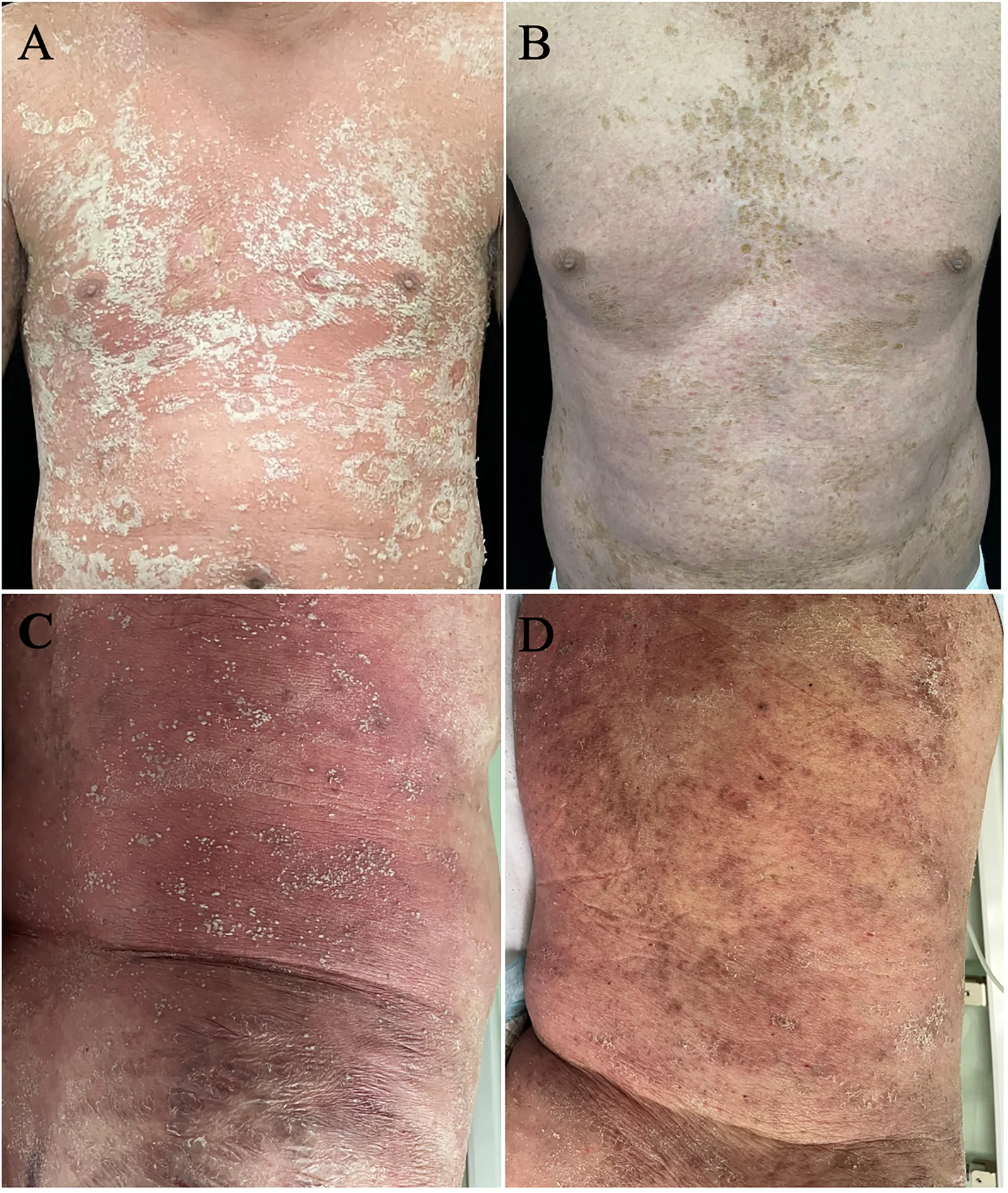
Improvement of skin lesions in two generalized pustular psoriasis (GPP) patients before and after spesolimab therapy. Pre-treatment [Case 1: **(A)** Case 2: **(C)**]: Two patients presented with typical GPP-related erythema and pustular rashes. Post-treatment [Case 1: **(B)**, Case 2: **(D)**]: In both cases, skin erythema and pustules were significantly relieved and the skin lesions were markedly improved after spesolimab treatment, demonstrating the rapid therapeutic efficacy of spesolimab for acute GPP flares.

Past medical history: Notable for chronic renal insufficiency.

Physical examination: Revealed extensive edematous erythema with numerous pinhead-sized sterile pustules and areas of confluent pustulation (“lakes of pus”) on the trunk and limbs.

Disease assessment: GPP Area and Severity Index (GPPASI) score (12) was 35. Numeric Rating Scale (NRS) for pain was 3, and Visual Analogue Scale (VAS) for pruritus was 8.

### 2.2 Case 2

A 61-year-old female was admitted in our hospital with a 3-day history of acute pustular lesion exacerbation (**Figures 1C, D**). She reported a 20-year history of psoriasis with recurrent pustular transformation. Over the past two decades, ingestion of allergenic foods had triggered multiple episodes of pustules superimposed on pre-existing rash. During significant exacerbations, the rash was managed with subcutaneous corticosteroid injections (specific timing and dosage unknown), discontinued after control was achieved.

Past medical history: Included chronic renal insufficiency, hypertension, and type 2 diabetes mellitus.

Physical examination: Demonstrated diffuse, ill-defined erythematous plaques and edematous patches with fine scaling on the face, trunk, and limbs, studded with numerous pinhead-sized sterile pustules. Grade 2 ascites was present.

Disease assessment: GPPASI score was 26.7, NRS was 3, and VAS was 7.

## 3 Investigations

### 3.1 Routine laboratory indicators

On admission, in Case 1, baseline serum creatinine was 117 μmol/L, with an estimated glomerular filtration rate of 61.32 calculated by the CKD-EPI creatinine equation. Urinalysis showed mildly elevated leukocytes (97/μL). The urine albumin-to-creatinine ratio was 91.02 mg/g (KDIGO category A2), and 24-hour urinary protein excretion reached 884.0 mg.

In case 2, emergency renal biochemistry showed serum creatinine 115 μmol/L. The estimated glomerular filtration rate calculated using the CKD-EPI creatinine equation was 44.18 . Urinalysis revealed severe pyuria (leukocytes 1097/μL), hematuria (erythrocytes 103/μL), and a small number of pathological casts. The urine albumin-to-creatinine ratio was 86.47 mg/g (KDIGO category A2), and 24-hour urinary protein excretion was 330.0 mg.

Both results met the diagnostic standard of moderate renal impairment in local clinical laboratory (13). Others, both CT and ultrasound of Case 1 indicated fatty liver with no other abnormalities detected. Liver function tests reveal a serum total protein level of 48.2 g/L and a serum albumin level of 27.1 g/L.

### 3.2 Skin histopathology

In Case 2 (**Figures 2A, B**), histopathological examination of a skin biopsy from the left forearm revealed hyperkeratosis, parakeratosis, and aggregates of neutrophils within the stratum corneum. Subcorneal pustule formation was noted, with marked edema of the dermal papillae. A perivascular infiltrate composed of lymphocytes and neutrophils was observed in the superficial dermis. These findings are consistent with a sterile pustular dermatosis and support the diagnosis of pustular psoriasis.

**Figure 2:**
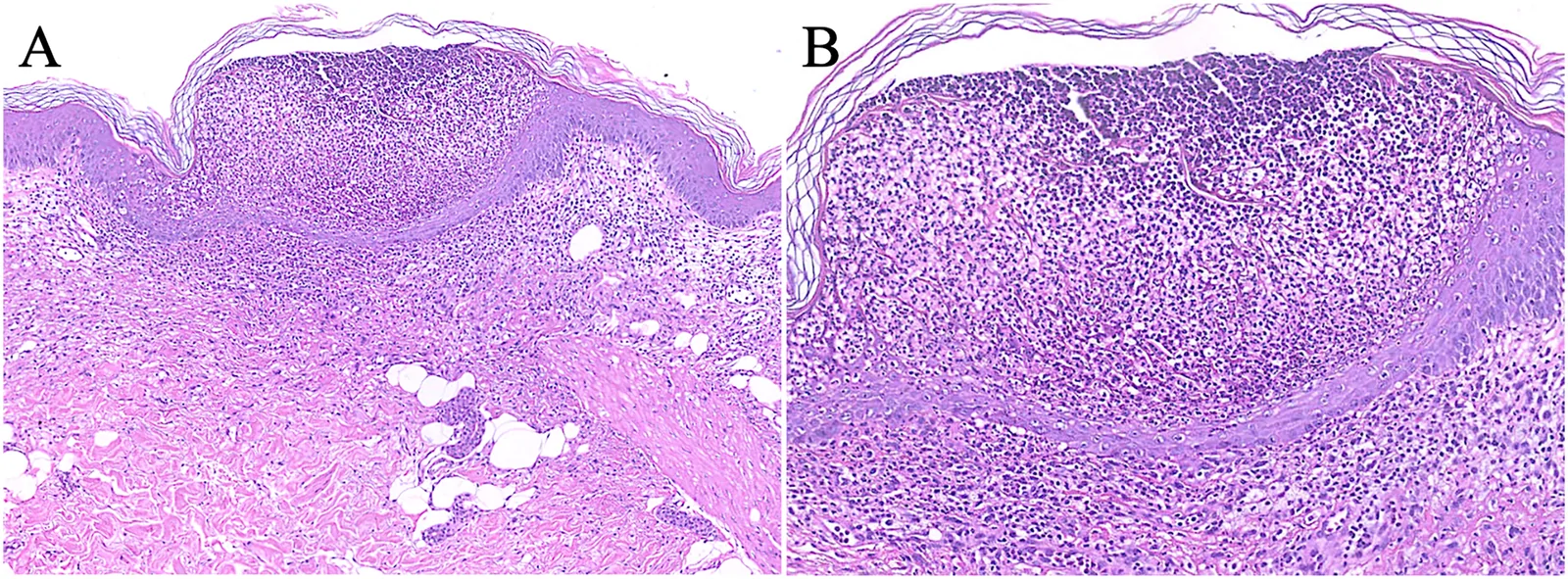
In Case 2, histopathological examination of a biopsy taken from a left forearm pustule revealed spongiform pustules in the epidermis and perivascular lymphocytic infiltration in the superficial dermis, consistent with pustular psoriasis **(A, B)**.

For Case 1, a repeat biopsy was not performed, as the diagnosis of GPP had already been confirmed by histopathology thirty years ago, and the patient had been followed up regularly in our hospital.

### 3.3 Serum inflammatory cytokines detection

Case 1: Serum was tested at a 1:1 dilution. The patient’s serum inflammatory cytokine levels were as follows: IL-2: 6.38 pg/mL, IL-4: 0.78 pg/mL, IL-6: 215.32 pg/mL, IL-10: 9.94 pg/mL, IL-17: 1.9 pg/mL, TNF-α: 7.36 pg/mL, IFN-γ: 1.12 pg/mL. IL-6 was markedly elevated, and IL-10 and TNF-α were also increased.

Case 2: Serum was tested at a 1:1 dilution. The patient’s serum inflammatory cytokine levels were as follows: IL-2: 13.14 pg/mL, IL-4: 3.74 pg/mL, IL-6: 180.66 pg/mL, IL-10: 14.5 pg/mL, IL-17: 10.16 pg/mL, TNF-α: 3.02 pg/mL, IFN-γ: 4.38 pg/mL. IL-6 was markedly elevated, and IL-10 and IL-2 were also increased.

### 3.4 Genetic testing detection

Case 1: Genetic testing was subsequently performed, revealing a homozygous mutation in the IL36RN gene (chr2:113060943–113060943 NM_012275.3: c.115 + 6T>C).

Case 2: Sequencing of the IL36RN gene was performed, and no pathogenic mutations were detected.

## 4 Treatment and outcome

Following comprehensive evaluation, both patients received a single 900 mg intravenous infusion of spesolimab. Supportive topical therapy with corticosteroids and emollients was concurrently administered. Clinical efficacy observation: Both patients demonstrated rapid clinical improvement within 24–48 hours, with pustules drying and erythema fading substantially. Within one week, GPPASI scores decreased by >90%. No infusion-related reactions or other adverse events were documented. Renal function parameters remained stable throughout the treatment period, which also indicated that both patients with GPP develop transient renal abnormalities during long-term immunosuppressive and biologic therapy. Additionally, the ascites markedly improved as the rash subsided.

Follow-up: Patients were followed up at 2 weeks, 1 month, 3 months, and 6 months post-treatment to assess efficacy and safety outcomes. Clinical assessments, including GPPASI scores and laboratory tests, were performed at each visit. Subsequent follow-up visits confirmed sustained remission without new pustule formation or disease flares. Both patients reported marked improvement in quality of life, and renal function parameters maintained stability.

## 5 Discussion

Spesolimab has transformed the therapeutic landscape for GPP (10). Its application in patients with significant renal impairment warrants particular attention, as traditional systemic agents such as cyclosporine often require dose adjustments and carry inherent renal risks (14). Spesolimab undergoes metabolism via protein catabolism, and its pharmacokinetic profile is not substantially influenced by renal function, rendering it a theoretically advantageous option for this population (15). The two cases presented here contribute to the growing evidence supporting the efficacy and safety of spesolimab in the rapid control of acute flares of GPP. Notably, both patients achieved a dramatic and sustained clinical response despite episodes of transient renal insufficiency, a high-risk comorbidity that is often a source of concern when initiating novel biologic therapies.

The central role of the IL-36 signaling pathway in the pathogenesis of GPP is well-established, and spesolimab’s mechanism of action directly targets this key inflammatory driver. Interestingly, in our report, while Case 1 harbored a confirmed IL36RN mutation, Case 2 was negative for IL36RN mutations. This is consistent with previous reports indicating that not all GPP patients carry identifiable IL36RN mutations (16). Despite the genetic heterogeneity, both patients achieved rapid pustular clearance with spesolimab, suggesting that IL-36 pathway inhibition is effective regardless of the IL36RN mutational status (17). Moreover, the rapid clinical improvement observed in our patients—with pustule resolution and erythema fading within 24–48 hours and a >90% reduction in GPPASI score within one week—aligns with pivotal trial data, reinforcing its role as a highly effective first-line biologic for severe flares (10). Therefore, this supports the hypothesis that IL-36 pathway hyperactivation is the core pathogenic driver in GPP, regardless of the mutational status, and that IL-36 blockade is an effective therapeutic strategy for both mutant and wild-type patients.

The systemic symptoms and high inflammatory markers (CRP, IL-6) seen in our patients are characteristic of the IL-36-mediated inflammatory cascade. IL-36 acts as a potent inducer of downstream proinflammatory cytokines, including IL-6 and TNF-α (18). Although serum IL-36 levels were not measured in this study, to clarify the pathogenic driver, we conducted genetic testing. The identification of an IL36RN mutation in Patient 1 confirms the central role of the IL-36 pathway in this case. Therefore, the marked elevation of serum IL-6 and TNF-α observed in both patients likely reflects the systemic inflammatory response driven by the IL-36 axis, consistent with the pathophysiology of GPP. Spesolimab effectively quelled this inflammation (19), confirming that blocking the IL-36 receptor is a valid therapeutic approach for both mutant and wild-type GPP presentations. For Patient 2 with the absence of an IL36RN mutation (wild-type), the similar cytokine profile suggests that the IL-36 axis may still be activated via other regulatory mechanisms. Regrettably, we were unable to assess post-treatment inflammatory markers due to retrospective study design.

It is also noteworthy that Case 1 had a suboptimal response to prior ustekinumab (an IL-12/23 inhibitor) therapy, achieving only partial control before the acute flare precipitated by an antibiotic. This highlights the distinct pathophysiology of GPP compared to plaque psoriasis and suggests that targeting the IL-23/Th17 axis (as with ustekinumab) may be insufficient for some patients with severe IL-36-driven pustular disease (20). The subsequent complete and rapid response to spesolimab in underscores the importance of selecting a therapy that aligns with the dominant pathogenic pathway. It is noteworthy that systemic corticosteroids are generally used with caution in GPP. While subcutaneous corticosteroid injections were administered to both patients in our cohort, which provide rapid symptomatic relief for severe, localized, and painful lesions while awaiting the full therapeutic effect of spesolimab. This approach was taken as a supportive measure to manage acute local symptoms.

The primary clinical significance of these cases lies in their demonstration of spesolimab’s tolerability in patients with concomitant moderate renal insufficiency (21). Patients with renal insufficiency represent a vulnerable population often excluded from or underrepresented in clinical trials. Concerns regarding altered drug pharmacokinetics, potential nephrotoxicity, or increased susceptibility to infections could complicate therapeutic decisions. In our patients, renal function remained stable, and no adverse events, including infusion reactions or infections, were observed. This illustrates that a single standard dose of spesolimab was both highly effective and well-tolerated, achieving rapid pustule clearance without exacerbating renal dysfunction. These findings suggest spesolimab is a valuable therapeutic alternative for GPP patients with comorbid renal insufficiency, a population posing complex management challenges. The absence of adverse events aligns with established safety data and the drug’s metabolic pathway. While spesolimab showed rapid efficacy and short-term safety in patients with renal insufficiency, long-term prospective studies are needed to fully evaluate its impact on renal function and renal insufficiency progression over time.

## 6 Conclusion

In conclusion, these cases provide real-world evidence that spesolimab is a rapidly effective and well-tolerated treatment for acute GPP flares, even in patients with moderate renal insufficiency. Its use led to swift pustule clearance, significant symptom relief, and sustained remission without compromising renal safety in our short-term observation. Further studies with larger cohorts and longer follow-up durations are needed to confirm the long-term safety and efficacy of spesolimab, particularly in patients with significant comorbidities like renal insufficiency Nevertheless, these findings support the consideration of spesolimab as a viable and potent therapeutic option for this severe dermatological emergency across a broader patient population.

## 7 Patient perspective

Both patients were offered the opportunity to provide a statement regarding their care and experience but declined to do so. This is a common limitation in clinical documentation, as patient participation in this aspect of record-keeping is voluntary. The absence of a direct patient perspective is noted for completeness. The clinical assessment and plan of care detailed in this report are based on the available medical history, physical examination findings, and diagnostic studies.

## Data Availability

The raw data supporting the conclusions of this article will be made available by the authors, without undue reservation.
